# Cysteine String Protein Limits Expression of the Large Conductance, Calcium-Activated K^+^ (BK) Channel

**DOI:** 10.1371/journal.pone.0086586

**Published:** 2014-01-27

**Authors:** Eva Ahrendt, Barry Kyle, Andrew P. Braun, Janice E. A. Braun

**Affiliations:** 1 Department of Physiology and Pharmacology, Hotchkiss Brain Institute, University of Calgary, Calgary, Alberta, Canada; 2 Libin Cardiovascular Institute, Faculty of Medicine, University of Calgary, Calgary, Alberta, Canada; University of Toronto, Canada

## Abstract

Large-conductance, calcium-activated K^+^ (BK) channels are widely distributed throughout the nervous system and play an essential role in regulation of action potential duration and firing frequency, along with neurotransmitter release at the presynaptic terminal. We have previously demonstrated that select mutations in cysteine string protein (CSPα), a presynaptic J-protein and co-chaperone, increase BK channel expression. This observation raised the possibility that wild-type CSPα normally functions to limit neuronal BK channel expression. Here we show by Western blot analysis of transfected neuroblastoma cells that when BK channels are present at elevated levels, CSPα acts to reduce expression. Moreover, we demonstrate that the accessory subunits, BKβ4 and BKβ1 do not alter CSPα-mediated reduction of expressed BKα subunits. Structure-function analysis reveals that the N-terminal J-domain of CSPα is critical for the observed regulation of BK channels levels. Finally, we demonstrate that CSPα limits BK current amplitude, while the loss-of-function homologue CSPα_HPD-AAA_ increases BK current. Our observations indicate that CSPα has a role in regulating synaptic excitability and neurotransmission by limiting expression of BK channels.

## Introduction

Cysteine string protein (CSPα) is a presynaptic co-chaperone for Hsc70 that protects neurons from degeneration and synaptic loss [1]. CSPα is a synaptic vesicle-associated protein bearing a characteristic J domain, as well as a cysteine rich ‘string’ region [2]. Mutations of CSPα in humans are associated with adult-onset autosomal dominant neuronal ceroid lipofuscinosis (ANCL), which is a progressive neurodegenerative disorder characterized by psychiatric manifestations, seizures, progressive dementia and motor impairment [3]–[5]. Disruption of the CSPα gene causes impaired presynaptic neurotransmission in *Drosophila melanogaster* [6] and fulminant neurodegeneration in mice [1], [7]. In CSPα null mice, synapse loss occurs in an age- [1] and activity-dependent manner [8], [9]. The cellular mechanisms that underlie CSPα’s neuroprotective function remain to be established.

Recent work from our group has shown that the large-conductance, calcium-activated K+ (BK) channel, which plays an important role in neuronal membrane excitability, is markedly increased by disabling mutations of CSPα [10]. Specifically, deletion of CSPα residue 116, replacement of Leu 115 by Arg lead to increased BK channel density in cultured neurons. Moreover, CSPα null mice exhibit 2.5 fold higher BK channel expression compared to wild type mice, whereas the densities of other important cation channels (i.e. Ca_v_2.2, K_v_1.1 and K_v_1.2) do not change. These findings suggest that one particular function of CSPα may be to limit the cellular expression of BK channels.

In the present study, we extend our recent work and characterize the effect of wild type CSPα on BK channel density. In particular, we have examined the prediction that wild-type CSPα limits the cellular level of BK channels, however, such an effect may only be evident under conditions of elevated BK channel expression. To create such a model system, murine neuroblastoma cells were transfected with murine BK channel cDNA in either the absence or presence of co-transfected wild-type, CSPα, or loss-of-function CSPα_HPD-AAA_. Using this strategy, we observed that CSPα limits BK channel expression in a time- and dose-dependent manner, and that the J domain of CSPα is essential for this regulatory action, as revealed by structure-function analysis. Finally, we show that while CSPα reduces BK channel current, loss-of-function CSPα_HPD-AAA_ increases BK current amplitude. These findings thus demonstrate that wild-type CSPα is able to restrict neuronal BK channel expression, and further help to explain why loss of CSPα function, due to genetic mutations, lead to elevated BK density in the CNS.

## Results

### Robust BK Channel Expression is Reduced by CSPα

**Figure 1:**
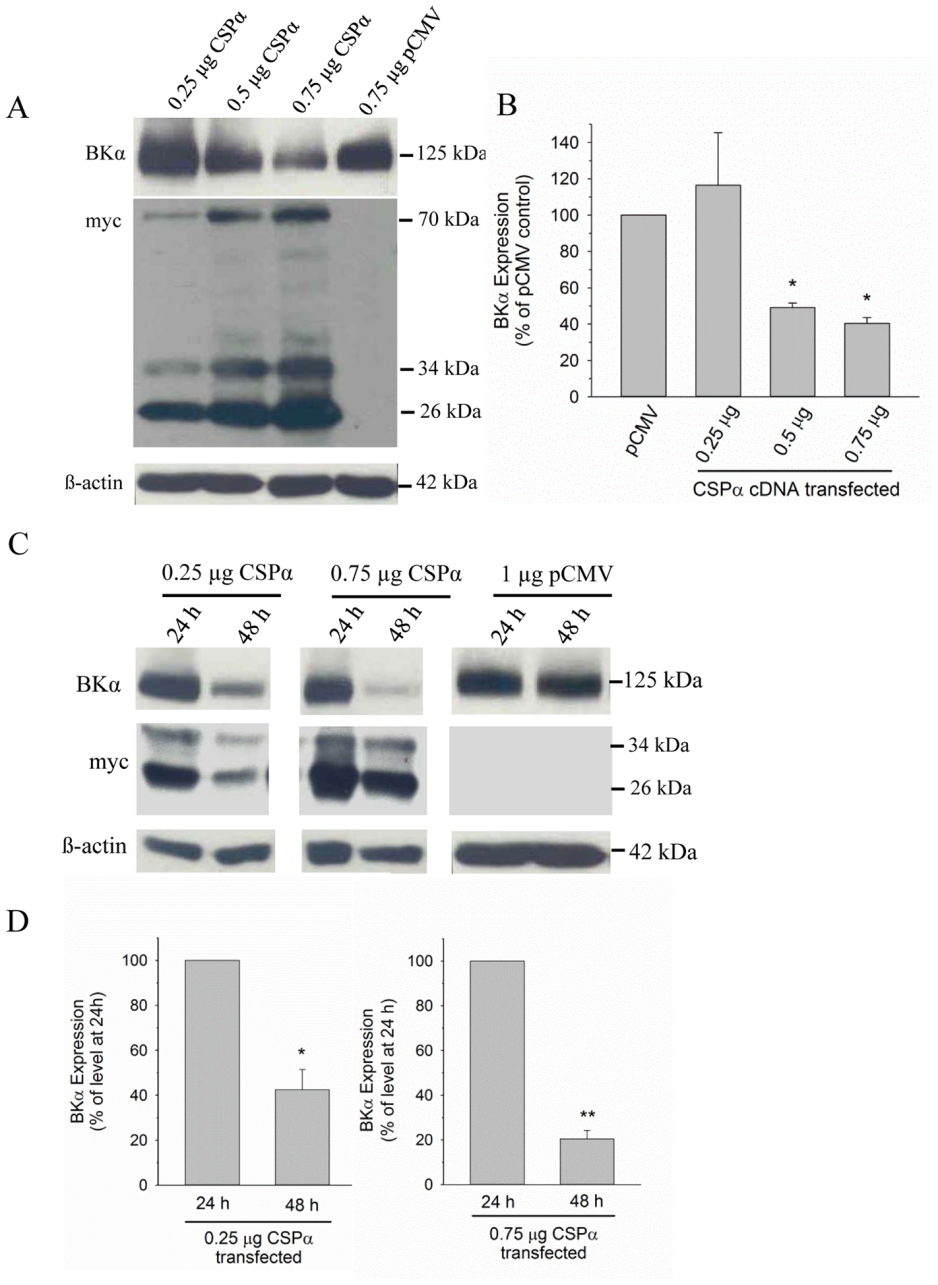
CSPα alters BK channel expression. **A.** Native CAD cells were transiently transfected with 1 µg cDNA encoding a neuronal BKα subunit, along with different amounts of myc-tagged CSPα (0.25 µg, 0.5 µg and 0.75 µg). Empty pCMV expression vector (0.75 µg) was co-transfected with 1 µg BKα subunit cDNA as a transfection control. 24 h post-transfection, the cells were lysed and the expression of BKα subunit and myc-tagged protein was analyzed by Western Blot. β-actin detection is shown to verify comparable sample loading. **B.** Histogram depicting quantification of BKα subunit levels in CAD cells co-transfected with increasing amounts of CSPα cDNA. Data are presented as mean ± SE of 5 similar experiments; *p<0.05 vs. pCMV vector control. **C.** Cells were transfected with 1 µg cDNA encoding BKα subunit along with either 0.25 µg or 0.75 µg of myc-tagged CSPα or 1 µg of pCMV. 24 h and 48 h post-transfection, BKα subunit expression was analyzed by Western Blot. **D.** Histograms depicting quantification of immunoreactive BKα subunit observed in the presence of co-transfected CSPα, as displayed in panel C. BKα subunit immunoreactivity detected at 48 h is expressed relative to the level of BKα subunit observed at 24 h; data are presented as mean ± SE of 4 similar experiments. Statistical significance was determined using one way ANOVA, *p<0.05; **p<0.01.

We have recently reported that interference of CSPα activity, either by genetic disruption (i.e. CSPα^−/−^ mice) or expression of dysfunctional CSPα in a neuronal cell line, is associated with a significant elevation of BK channel density at the cell surface [10]. The data arising from the experimental strategies designed to disrupt CSPα function strongly suggest that part of CSPα’s normal function in the CNS may be to regulate neuronal BK expression, which would be expected to influence neuronal excitability. We rationalized that if CSPα truly acts in this capacity, then a strategy involving elevated expression of wild-type CSPα would predictably lead to a decrease in BK channel levels, and provide direct insights into the cellular actions of wild-type CSPα. To test this hypothesis, we utilized a transient transfection strategy in order to express murine brain BK channel α subunits at a high level, thereby providing a robust baseline signal from which one could reliably detect hypothesized decreases in BK channel levels in the presence of increasing amounts of wild-type CSPα. As shown in Figure 1A, co-expression of murine brain BKα subunits (Butler et al, 1993) in native CAD cells with increasing amounts of myc-tagged, wild-type CSPα led to a dose-dependent decrease in the cellular level of BKα subunit protein, which correlated with increasing cellular expression of CSPα. Whereas co-transfection of cells with a low amount of CSPα cDNA (i.e. 0.25 µg) had no significant effect on BKα channel expression at 24 hour post-transfection, addition of either 0.5 µg or 0.75 µg CSPα cDNA significantly reduced the level of BKα protein. Figure 1B displays quantification of the CSPα-dependent changes in BKα subunit expression; data are normalized to the level of BKα subunit in the presence of empty pCMV expression vector, which served as the co-transfection control. These experiments revealed that wild-type CSPα is capable of decreasing BK channel density in a dose-dependent manner.

We next examined the time dependence of the observed CSPα-mediated regulation of BK channel expression. Figures 1C&D show that the extent of CSPα-mediated decrease in BKα subunit expression was greater at 48 hours post-transfection using either 0.25 or 0.75 µg CSPα cDNA compared with the expression observed at 24 hours. The greater effect of CSPα on BK channel expression at 48 hours was not due to enhanced expression of CSPα at the 48 versus 24 hour time point, as shown by anti-myc detection (Figure 1C). To quantify the greater effect of CSPα at 48 hours, we compared BK channel expression at this time point with the expression at 24 hours, which was first normalized to 100% for both 0.25 and 0.75 µg CSPα transfection conditions (Figure 1D). With a higher level of CSPα expression (i.e. 0.75 vs 0.25 µg), a more substantial decrease in BK channel expression at the 48 hour time point was noted. No CSPα-mediated changes were detected in cellular level of β-actin and co-transfection with the pCMV vector alone did not result in any time-dependent alteration of BK channel density. The data displayed in Figure 1 further illustrate the 3 distinct species of wild-type CSPα that can be identified by Western blot; a 26 kDa immature form, a 34 kDa mature palmitoylated protein and a 70 kDa CSPα dimer, as described previously [11], [12]. Taken together, these findings show that the wild-type CSPα is able to lower BKα subunit expression in a dose- and time-dependent manner.

**Figure 2:**
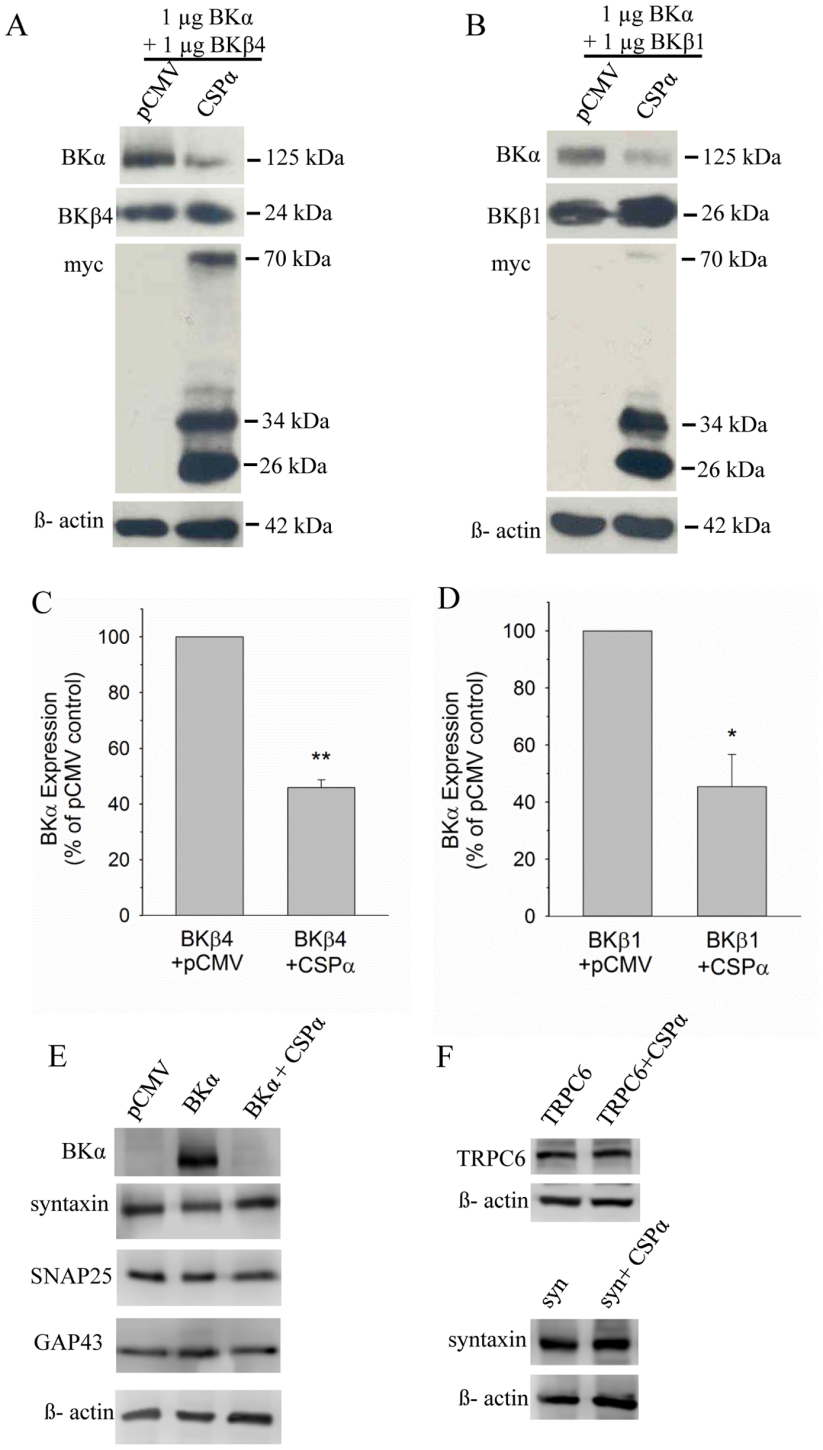
BK channelβ-subunits (BKβ4 and BKβ1) do not alter the CSPα-mediated reduction in BK channel expression. CAD cells were transiently transfected with 1 µg BKα subunit cDNA plus 1 µg BKβ4 subunit cDNA (**A**) or 1 µg BKβ1 subunit cDNA (**B**), along with either 0.75 µg CSPα or pCMV vector (control). 24 h post-transfection, protein expression was analyzed by Western Blot with primary antibodies recognizing BKα subunit, BKβ4 subunit, BKβ1 subunit, myc epitope tag, and β-actin. **C** and **D,** Quantification of BKα subunit expression in CAD cells in the absence or presence of co-transfected CSPα, along with the presence of either BKβ4 (panel C) or BKβ1 (panel D). **E** CAD cells were transfected with cDNAs encoding pCMV vector alone, BKα subunit or BKα subunit+CSPα and the indicated proteins were evaluated by Western blot analysis. **F** CAD cells were transfected with TRPC6 channel or syntaxin1A cDNA in either the absence of presence of CSPα cDNA. Detection of β-actin was utilized to verify equal protein sample loading. Data are presented as mean ± SE of 3 similar experiments; statistical significance was determined using one way ANOVA, *p<0.05; **p<0.01. Data shown in E and F are representive of 4 independent experiments.

Physiologically, BKα subunits co-assemble into a tetrameric complex with a single, ion conducting pore structure that is subject to regulation by auxiliary β-subunits. Since chaperones typically regulate the assembly and/or disassembly of protein complexes (e.g. DnaJC6 mediates clathrin disassembly [13]), we investigated the possibility that the presence of auxiliary BKβ-subunits may alter the observed CSPα-mediated regulation of BK channel expression. As shown in Figure 2, co-expression of the BK channel auxiliary subunits, BKβ4 (panel A) or BKβ1 (panel B), did not influence the CSPα-mediated decrease in BK channel levels in transfected CAD cells. Co-expression of CSPα still reduced BK channel levels in the presence of BKβ1 or BKβ4, compared to vector control. Interestingly, CSPα did not noticeably alter the expression of either BKβ1 or BKβ4 (Fig. 2A and B, middle panels). In CAD cells transfected with BKα cDNA in either the absence or presence of CSPα, we further observed that the expression levels of several endogenous membrane proteins (i.e. SNAP25, syntaxin1A and GAP43) were not altered (Figure 2E). Moreover, we found that co-transfected CSPα does not alter the expression of the membrane proteins syntaxin 1A or the TRPC6 channel isoform following their transient expression in CAD cells (Figure 2F). Collectively, these results indicate that BKβ1 and BKβ4 do not influence the regulation of BK channel expression by CSPα and that CSPα selectively reduces BKα subunit expression, but not that of either BKβ1 or BKβ4 or a number of other membrane proteins.

### The J Domain is Essential for CSPα-mediated Reduction in BKα Channels

**Figure 3:**
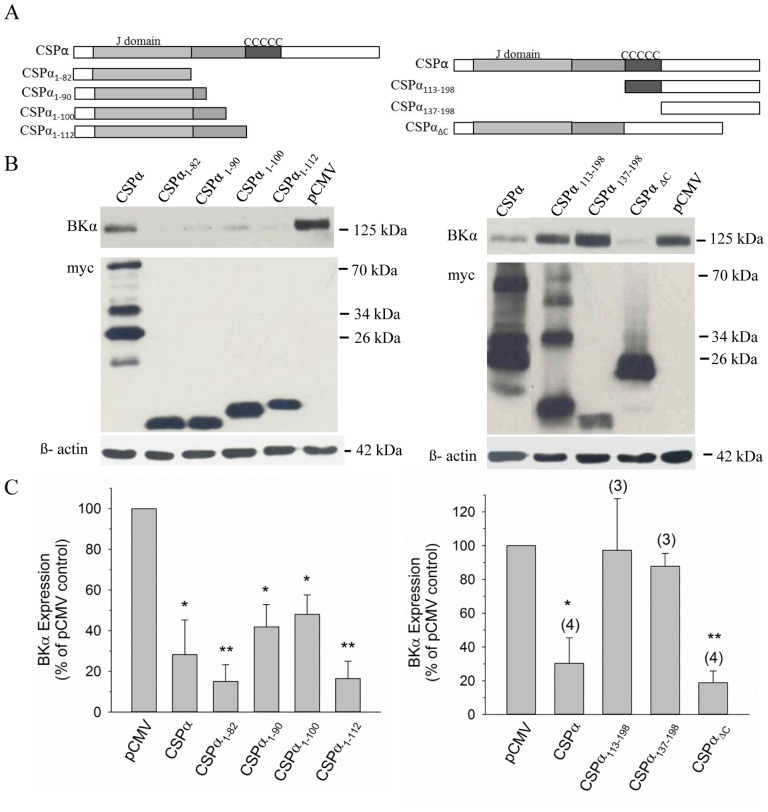
The J domain of CSPα reduces BK channel expression. **A.** Schematic of myc-tagged full length CSPα and CSPα deletion constructs. **B.** Western blot analysis of BK channel expression in CAD cells 24 h post-transfection with 1 µg cDNA encoding BKα subunit along with 0.75 µg myc-tagged full length CSPα cDNA or the indicated deletion constructs. As a transfection control, 0.75 µg empty pCMV was co-transfected with BKα subunit cDNA. 30 µg of cell lysate isolated under each experimental condition was separated by SDS-PAGE, probed with an anti-BKα subunit antibody and an anti-myc antibody. The histograms in panel **C** quantify changes in BK channel expression in the presence of wild-type CSPα and individual CSPα deletion mutants. Statistically significant differences from the pCMV control (set to 100%) were determined by one-way ANOVA; *p<0.05; **p<0.001.

To elucidate the structural elements within CSPα responsible for its regulation of BK channel expression, a series of CSPα deletion mutants were constructed and co-transfected with BKα subunit cDNA in CAD cells. The myc-tagged CSPα constructs are shown schematically in Figure 3A, and each cDNA construct generated a protein that migrated at the expected molecular weight when analyzed by SDS PAGE and western blotting (Fig. 3B). Experimentally, all C-terminal truncations of CSPα were still capable of decreasing BK channel expression following co-transfection. CSPα_1–90_ and CSPα_1–100_ reduced BK channel expression to levels of 41.9±10.9% and 48.1±9.5% of control, respectively, and larger reductions in channel expression were observed in the presence of CSPα_1–82_ (15.1±8.2% of control) and CSPα_1–112_ (16.5±8.6% of control), relative to cells co-transfected with the pCMV vector (Fig. 3C). These findings indicate that the N-terminal region of CSPα, which contains the J domain, is sufficient for the observed regulation of BK channel expression by wild-type CSPα. This conclusion was further confirmed by examining N-terminal truncations of CSPα lacking the J domain. Neither CSPα_113–198_ (97.3±30.5%) nor CSPα_137–198_ (87.3±7.6%) had a significant effect on BK channel expression (Fig. 3C), emphasizing the importance of the N-terminal region of CSPα for the regulation of BK channel expression. As expected, multiple bands are observed for CSPα_113–198_ which includes the cysteine string region, while a single immunoreactive band was detected for CSPα_137–198_ which does not include the cysteine string region. We have previously demonstrated that residues 83–136, encoding the linker region and cysteine string region are required for CSPα oligomerization [11] as exemplified in the right hand side of Figure 3B. Interestingly, deletion of the cysteine string region (CSPα_ΔC_) did not preclude the ability of CSPα to reduce BK channel expression (18.8±7.0%).

**Figure 4:**
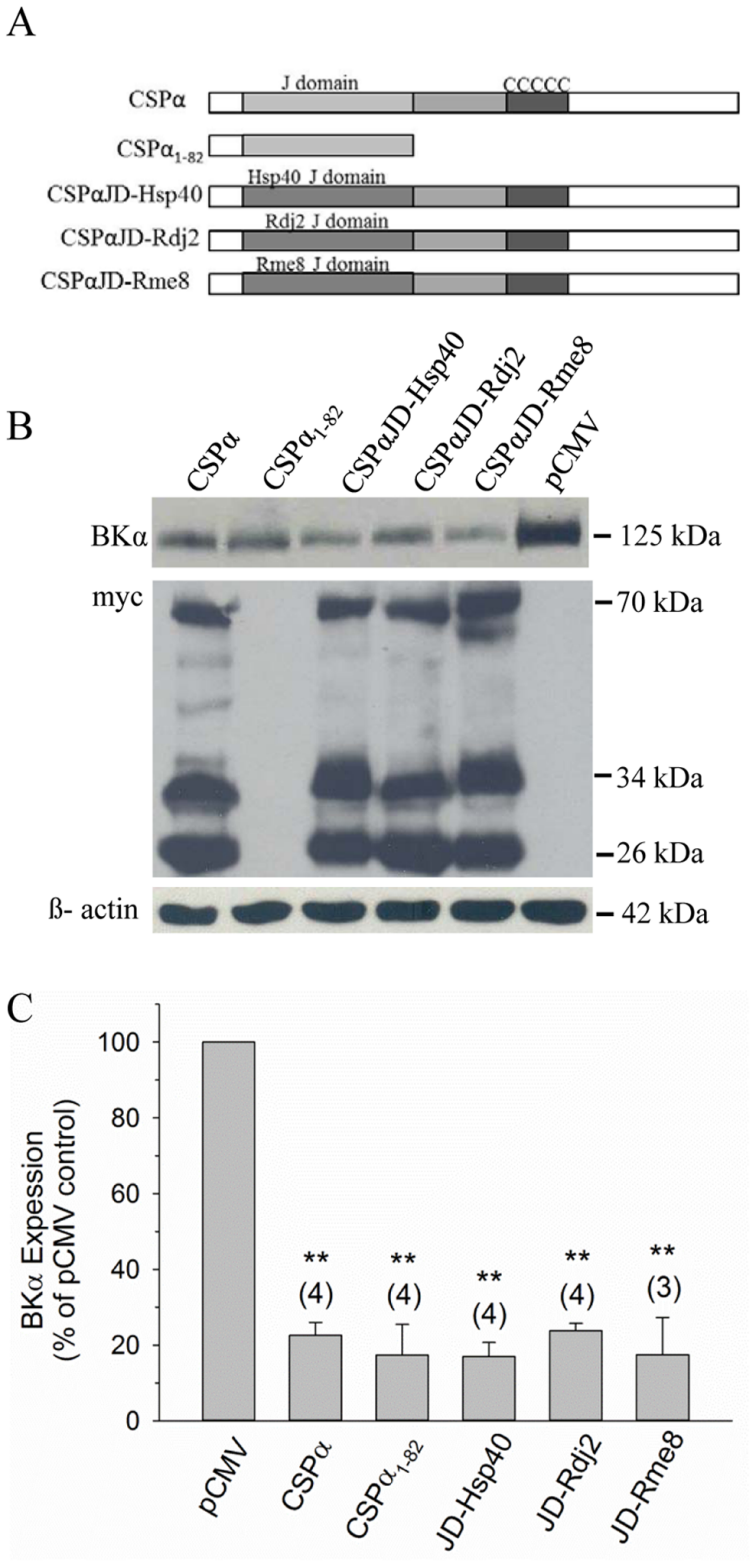
CSPα chimeras with substituted J domains alter BK channel expression. **A.** Schematic of wild-type CSPα and CSPα chimeras in which the J domain of CSPα is replaced by the J domain of the related J proteins Hsp40, Rdj2 or Rme8. **B.** Western blot analysis of BK channel expression in CAD cells 24 h following transfection with cDNA encoding BKα subunit and myc-tagged CSPα or the indicated CSPα myc-tagged chimeric constructs. 0.75 µg of pCMV vector was co-transfected with 1 µg cDNA encoding BKα subunit as a transfection control. Expression of myc-tagged proteins is shown by western blot, along with β-actin detection to verify similar sample loading. **C.** Histogram showing quantification of BK channel expression in the presence of either wild-type or J-domain (JD) substituted CSPα chimeras relative to cells co-transfected with pCMV vector. Mean data were obtained from 3–4 independent experiments, and statistically significant differences were determined by one-way ANOVA; **p<0.001.

Several neuronal proteins contain a J-domain [14], which represents the signature motif of all members of the J protein family. Given the functional importance of the J-domain for CSPα’s observed regulation of BK channel levels, we asked whether J-domains from related chaperones were sufficiently conserved to substitute in this process. To address this question, we generated chimeras of CSPα in which the native J-domain was replaced by the J-domain from another J protein chaperone. The resulting chimeric constructs, shown schematically in Figure 4A, consist of a CSPα background and a substituted J-domain obtained from Hsp40 (DnaJB1), Rdj2 (DnaJA2) and Rme8 (DnaJC13), which display 52%, 52% and 44% amino acid identity, respectively, with the J-domain of CSPα (rat isoform). Western blot analysis demonstrated that co-transfection with individual CSPα/J-domain chimeras decreased BK channel expression compared to the pCMV vector (Figure 4B). Quantification of these effects revealed that the CSPα chimeras - CSPαJD_Hsp40_ (17.0±3.7%), CSPαJD_Rdj2_ (23.8±2.0%) and CSPαJD_Rme8_ (17.5±9.8%), along with wild-type CSPα (22.6±3.4%) and the truncation mutant CSPα_1–82_ (17.4±8.1%), all produced a statistically significant and comparable reduction in BK channel expression. As depicted in Figure 4B, all three CSPα chimeric constructs expressed to a similar level compared with wild-type CSPα, and three distinct molecular species of wild-type and chimeric CSPα isoforms (i.e. 26 kDa, 34 kDa and 70 kDa) were readily identified by Western blot, regardless of the substituted J-domain. (Note that the CSPα_1–82_ construct was not detected on this blot, due to its rapid migration during SDS-PAGE. However, it is evident from Figure 3B that this construct expresses well under our experimental conditions). As displayed in preceding figures, β-actin staining was utilized to ensure similar protein loading for the various cell lysates. Based on these data, it appears that the J-domain of CSPα is necessary for the regulation of BK channel expression and that individual J-domains from related J protein family members can functionally substitute for the native J-domain in CSPα.

**Figure 5:**
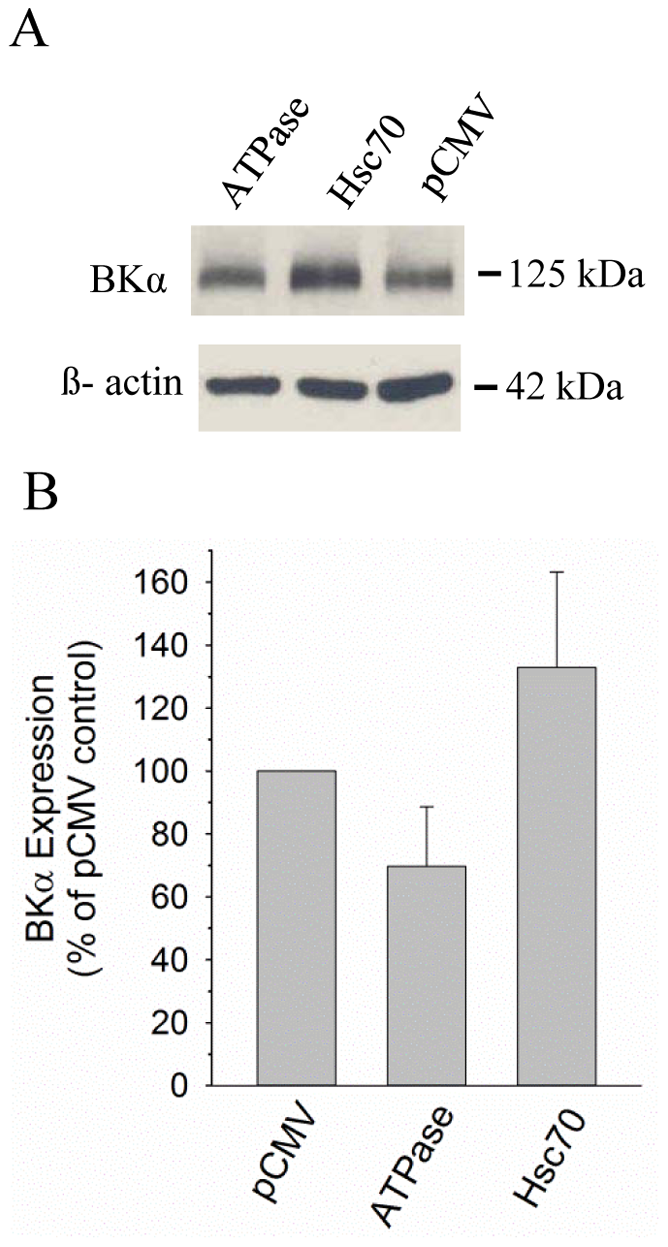
Hsc70 does not reduce BK channel expression in the absence of CSPα. **A.** CAD cells were transiently co-transfected with 1 µg cDNA encoding BKα subunit and either 1 µg HA-tagged, wild-type Hsc70 or the Hsc70 ATPase domain. Empty pCMV vector (1 µg) was co-transfected with 1 µg cDNA encoding BKα subunit as a transfection control. 24 h post-transfection, the cells were lysed and BK channel and β-actin expression was analyzed. **B**. The histogram quantifies the relative changes in cellular BK channel expression in the presence of WT Hsc70 and the Hsc70 ATPase domain, which were not statistically different than the control (i.e. pCMV vector alone).

Hsc70 is a critical interacting partner of CSPα, and displays a low basal ATPase activity that is activated following interaction with the J-domain of CSPα [15]. We examined the possibility that over-expression of Hsc70 alone may be able to independently evoke a reduction in BK channel levels, similar to that observed for CSPα. CAD cells were transiently co-transfected with cDNA encoding the BKα subunit, along with either HA-tagged, wild-type Hsc70, the HA-tagged ATPase domain of Hsc70, which displays constitutive activity [15] or pCMV vector (negative control). Figure 5 demonstrates that no significant changes in BK channel expression were observed when either full length Hsc70 (132.9±30.3%) or the active ATPase domain (i.e. Hsc70_1–386_) of Hsc70 (69.7±18.9%) was co-expressed with the BKα subunit, compared with pCMV vector alone. These data indicate that increased cellular levels of Hsc70 are not sufficient to regulate BK channel expression and that activation of Hsc70 by CSPα is likely required. Our observations are thus similar to those described by Walker and colleagues, who reported that DnaJA1, DnaJA2 and DnaJA4 reduced hERG channel maturation, whereas over-expression of Hsc70 alone had no effect on maturation events [16].

### BK Channel Current Density is Decreased by CSPα

**Figure 6:**
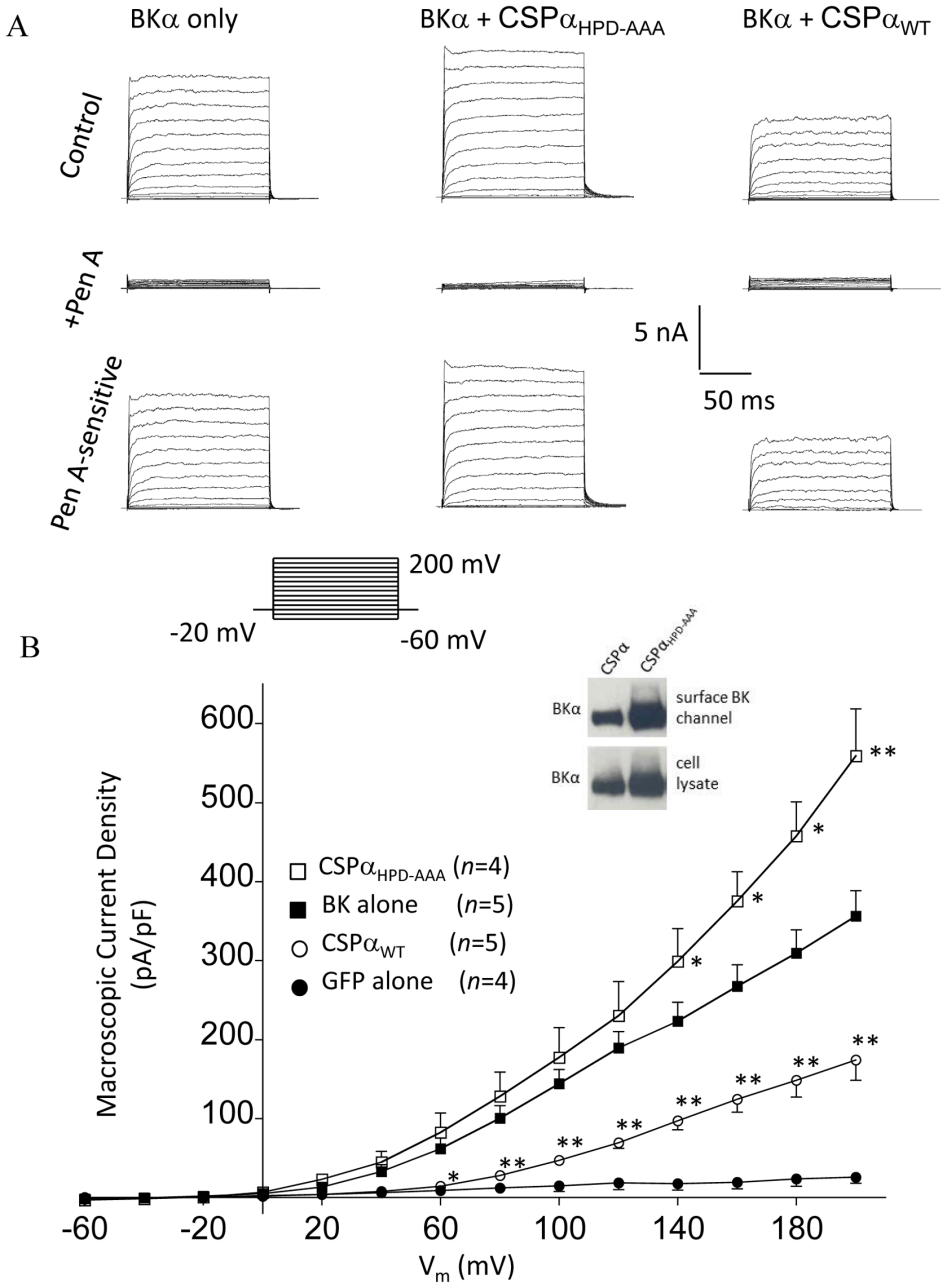
CSPα co-expression alters BK channel whole cell current density in transiently transfected CAD cells. (A) Representative recordings of single CAD cells voltage clamped as depicted in the protocol using the whole-cell configuration of the patch clamp technique. The left-hand column displays macroscopic BK channel currents recorded from CAD cells transiently-transfected with BKα subunits alone. The middle and right-hand columns display BK currents recorded from cells co-transfected either a CSPα mutant (i.e. CSPα_HPD-AAA_) or wild-type CSPα, respectively. Recordings were done 40–48 hours post-transfection. In all three conditions, whole cell currents were largely inhibited following addition of penitrem-A (100 nM). Penitrem-A sensitive currents, obtained by digital subtraction, are displayed in the bottom row of traces. The vertical and horizontal scale bars shown apply to all current tracings. The current-voltage plot shown in panel B quantifies the average penitrem A-sensitive peak currents observed under each transfection condition and highlights the significant differences in current density magnitude found between CAD cells transiently-transfected with BKα subunits alone, and those co-transfected with either WT or a mutant form of CSPα (Dunnett post-hoc ANOVA, one-way Vs BK channel alone; *p<0.05, **p<0.01). Inset shows BK channel expression in CAD cells transiently co-transfected with 0.75 µg cDNA encoding either CSPα or CSPα_HPD-AAA._ For detection of surface expressed BK channel, transfected CAD cells were labeled for 30 min at 4°C with bath applied Biotin, as described in the methods. Following lysis, 1 mg of soluble protein lysate was subjected to overnight streptavidin pull-down at 4°C. Proteins isolated by streptavidin pull down (top panel) and 30 µg of soluble cellular lysate (bottom panel) were analyzed by Western Blot with an anti-BKα subunit antibody. Data is representative of 3 similar experiments.

To determine whether the observed CSPα-mediated decrease in the cellular BKα protein level also reflected a reduction in functional BK channels at the cell surface, we carried out single cell patch clamp recordings of CAD cells transiently transfected with BKα subunit cDNA in the absence or presence of CSPα cDNA. Co-transfection of GFP under all conditions was utilized as a marker to identify transfected cells. Figure 6A shows representative current families recorded from transfected CAD cells expressing BKα subunit alone, or BKα along with either wild-type CSPα or the dysfunctional CSPα mutant CSPα_HPD-AAA_. Following control recordings, cells were treated with the highly selective BK channel blocker penitrem A to isolate BK channel currents. The bottom row of traces in Figure 6A display the magnitude of BK channel-mediated, penitrem A-sensitive current observed under the three different transfection conditions. The current-voltage plot displayed in Figure 6B quantifies the effect of CSPα co-expression on BK channel current density. In the presence of wild-type CSPα, BK current density was significantly decreased compared with BK channel alone, whereas co-transfection with the loss-of-function CSPα_HPD-AAA_ led to a higher current density at very positive test pulse voltages. In parallel experiments, these observed CSPα-mediated changes in cell surface BK channel density and were confirmed by cell surface biotin labeling, followed by streptavidin pull-down and Western analysis (refer to inset). We have previously reported that CSPα_HPD-AAA_ did not alter the expression of other membrane-associated proteins (e.g. GAP43 and flotillin) [10]. Taken together, these data demonstrate that CSPα limits the expression of functional BK channels at the cell surface.

## Discussion

We have found that CSPα, a presynaptic neuroprotective chaperone, acts to significantly limit BK channel current and BK channel expression. The CSPα-mediated reduction in BK channels is dose- and time- dependent and the N terminal J-domain of CSPα is essential for the noted decrease (Figures 1&3). Our earlier results reporting that loss/disruption of CSPα activity in either a knockout mouse or a neuronal cell line markedly increase BK channel expression [10] indirectly suggested that in the CNS, wild-type CSPα may function to limit cellular BK channel expression. In order to directly examine such an effect of wild-type CSPα, we utilized an expression strategy to force an imbalance between BK channels and endogenous CSPα, thereby allowing BK channel protein to be present at elevated levels so that decreases in BK channel density induced by CSPα could be precisely monitored. Physiologically, BK channel activity is regulated by multiple mechanisms, including modulatory accessory subunits [17], alternative splicing [18], phosphorylation [19] and palmitoylation [20]. Data presented here suggest that the CSPα-mediated reduction in BK channel density occurs in a graded fashion that is dependent upon the relative expression between BK channels and CSPα (i.e. when BK channel expression is high, the cellular level of CSPα must also be elevated in order for a reduction to occur). While BK channel expression was reduced by CSPα, at low expression levels of BK channels, the CSPα–mediated reduction was minimal. These data thus provide evidence that wild-type CSPα normally acts to restrict neuronal BK channel expression.

It is possible that the CSPα-induced regulation of BK channel current represents a key event contributing to the CSPα-mediated synapse protection. By influencing membrane excitability and action potential firing, abnormal BK channel current density could be the trigger for the cascade of events leading to neurodegeneration in ANCL patients and the fulminant neurodegeneration observed in CSPα-KO mice. Nonetheless, association studies have identified several other synaptic proteins that are also likely clients for CSPα and may contribute to the neuropathology associated with the loss/dysfunction of CSPα [21]–[28]. Trafficking proteins in CSPα controlled pathways include t-SNARE protein SNAP25 (synaptosomal associated protein of 25 kDa), which is required for exocytosis, the GTPase dynamin1, which is essential for endocytosis, and α-synuclein, which is implicated in synaptic vesicle function [7], [29]–[32]. It will therefore be important to determine whether the increased BK channel expression observed in our studies reflects a membrane trafficking defect involving one or more these putative pathways associated with CSPα activity and degeneration. While the importance of CSPα in synapse protection is well established, the series of events underlying protection and degeneration cascades remains to be determined.

In conclusion, we provide evidence that the presynaptic chaperone, CSPα, limits BK channel density. We further speculate that elevated BK channel expression may be involved in the severe age-dependent [1] and activity-dependent degeneration [8], [9] reported in the CNS and motor neurons of animal models displaying loss/dysfunction of CSPα [1], [8], [32], [33].

## Materials and Methods

### Materials

CAD (CNS catecholaminergic derived) mouse neuroblastoma cells [34] were seeded into 6 well plates and grown in DMEM/F12 medium supplemented with 10% fetal bovine serum and 1% penicillin/streptomycin, as previously described [12]. Cells were lysed in 40 mM Tris (pH 7.4), 150 mM NaCl, 2 mM EDTA, 1 mM EGTA, 1 mM Na_3_VO_4_, 0.1% SDS, 1% Triton X-100, 0.5 mM PMSF and protease inhibitor (Sigma) at 4°C for 1 hour. Lysates were centrifuged at 15000×g for 5 minutes at 4°C and the supernatant (soluble fraction) was collected and stored at −70°C. For transient transfection, CAD cells were washed in PBS and transiently transfected using Lipofectamine-2000 (Invitrogen) in Opti-MEM. Protein concentration of the soluble CAD cell fraction was determined using the Bradford assay (BioRad).

### Immunoblotting

Proteins (30 µg) were electro-transferred from SDS-polyacrylamide gels to nitrocellulose membrane (0.2 µm pore size) in 20 mM Tris, 150 mM glycine and 12% methanol. Membranes were blocked in phosphate-buffered saline (PBS) containing 0.1% Tween 20, 4% skim milk powder and then incubated with primary antibody overnight at 4°C. The membranes were washed and incubated with horseradish peroxidase-coupled secondary antibody. The signal was developed using West Pico reagent (Pierce Biotechnology Inc.) and exposed to Kodak x-ray film. Bound antisera were quantified using a Biorad Fluor-S MultiImager Max and QuantityOne 4.2.1 software.

### Whole Cell Patch Clamp Recordings

Voltage-clamp measurements were performed using conventional, ruptured membrane patch clamp methodology in combination with an Axopatch 200B amplifier, Digidata 1440 series analogue/digital interface and pClamp v10 software. Whole cell electrical signals were typically filtered at 1–2 kHz and sampled at 5 kHz. Glass micropipettes (2–4 MΩ tip resistance) were pulled from thin-walled borosilicate capillaries and were filled with a solution containing 100 mM KOH, 30 mM KCl, 1 mM MgCl_2_, 0.005 mM CaCl_2_, 10 mM HEPES, pH 7.3 with methanesulfonic acid. The bath chamber was placed on the stage of Nikon TE2000 inverted microscope equipped with epifluorescence illumination and perfused with a modified Ringer’s saline solution containing 135 mM NaCl, 5 mM KCl, 1 mM MgCl_2_, 2.5 mM CaCl_2_, 5 mM 4-aminopyridine and 10 mM HEPES, pH 7.3 with 1N NaOH. Cells in the bath chamber were constantly superfused at ∼2 ml/min and solution changes were performed by gravity flow from a series of elevated solution reservoirs using manually controlled solenoid valves. All electrophysiological recordings were performed at 35–37°C. CAD cells seeded on 35 mm sterile plastic dishes were transiently transfected with separate pcDNA3.1-based constructs encoding BKα subunit, wild-type or mutant CSPα. A separate cDNA construct encoding enhanced GFP was included in all conditions as a fluorescent marker of transfection. Cells were transfected for 5–6 hours using Lipofectamine 2000 as the transfection reagent, and the total amount of cDNA added per dish was typically 1.8–2 µg. Transfected cells were identified in the recording chamber by their green fluorescence using 488 nm excitation and 510 nm emission filters.

### Biotinylation of Cell Surface BK Channels

CAD cells transiently co-expressing murine brain BKα subunit [35] and CSPα variants were washed three times with PBS and incubated with EZ-Link Sulfo-NHS-SS Biotin (Thermo Scientific) (1 µg/ml) in PBS for 30 min at 4°C. As a negative control, cells were incubated only with PBS. The reaction was neutralized by addition of 1% (w/v) BSA in PBS for 10 min at 4°C. After neutralization, cells were washed with ice-cold PBS to remove non-reacted biotin, and were harvested in 1ml of PBS containing 1% v/v Triton X-100 and protease inhibitor (complete, EDTA-free, Sigma) by an incubation for 2–5 minutes on ice. The lysates were centrifuged at 15,000×g for 15 min at 4°C and the soluble protein concentration was determined using the Bradford assay (Bio Rad).

For streptavidin pull-down, 1 mg of the soluble protein lysate was incubated with 100 µl streptavidin agarose beads (50% slurry) (Thermo Scientific) overnight at 4°C on a rotator. Beads were centrifuged at 3.000×g and washed with 1% Triton X-100 in PBS. Following centrifugation, biotinylation proteins were eluted from the beads by adding 2× Laemmli sample buffer (62.2 mM Tris HCl pH 6.8, 7.5% v/v Glycerol, 2% w/v SDS, 0.015 mM Bromophenol Blue, 1.2% v/v β-Mercaptoethanol and 100 mM DTT) and incubated at 37°C for 1 h. Following elution, proteins were separated by SDS-PAGE.
