# Correction: Cysteine String Protein Limits Expression of the Large Conductance, Calcium-Activated K^+^ (BK) Channel

**DOI:** 10.1371/journal.pone.0140073

**Published:** 2015-10-02

**Authors:** Eva Ahrendt, Barry Kyle, Andrew P. Braun, Janice E. A. Braun

Fig 1C is incorrect as it shows the wrong actin blot. The authors have provided a corrected version of Fig 1 here.

**Fig 1:**
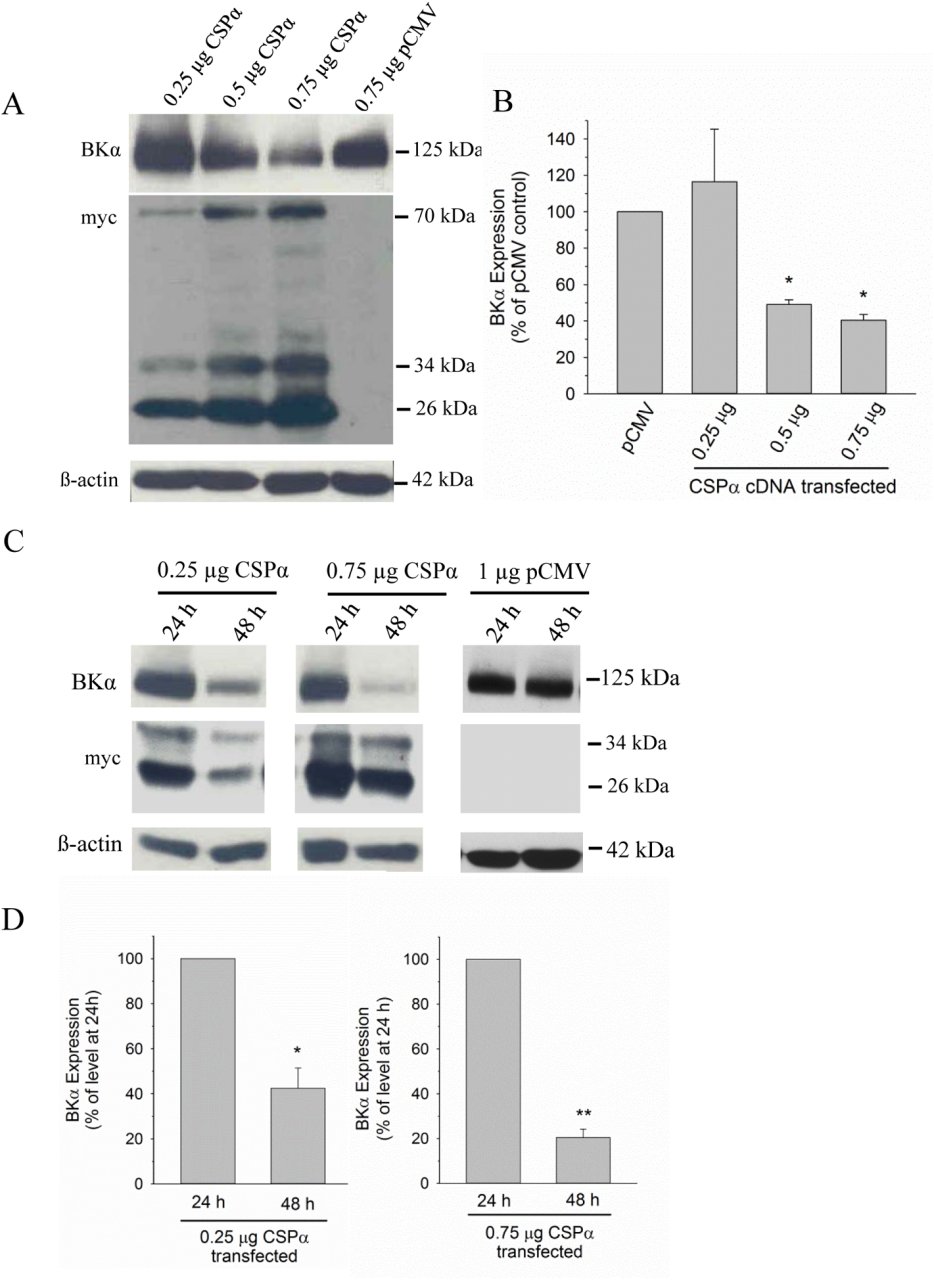
CSPα alters BK channel expression. **A.** Native CAD cells were transiently transfected with 1 μg cDNA encoding a neuronal BKα subunit, along with different amounts of myc-tagged CSPα (0.25 μg, 0.5 μg and 0.75 μg). Empty pCMV expression vector (0.75 μg) was co-transfected with 1 μg BKα subunit cDNA as a transfection control. 24 h post-transfection, the cells were lysed and the expression of BKα subunit and myc-tagged protein was analyzed by Western Blot. β-actin detection is shown to verify comparable sample loading. **B.** Histogram depicting quantification of BKα subunit levels in CAD cells co-transfected with increasing amounts of CSPα cDNA. Data are presented as mean ± SE of 5 similar experiments; *p<0.05 vs. pCMV vector control. **C.**Cells were transfected with 1 μg cDNA encoding BKα subunit along with either 0.25 μg or 0.75 μg of myc-tagged CSPα or 1 μg of pCMV. 24 h and 48 h post-transfection, BKα subunit expression was analyzed by Western Blot. **D.** Histograms depicting quantification of immunoreactive BKα subunit observed in the presence of co-transfected CSPα, as displayed in panel C. BKα subunit immunoreactivity detected at 48 h is expressed relative to the level of BKα subunit observed at 24 h; data are presented as mean ± SE of 4 similar experiments. Statistical significance was determined using one way ANOVA, *p<0.05; **p<0.01.

Additionally, there is a sentence missing from the caption for Fig 3. Please see the complete, correct Fig 3 caption here. The missing sentence is highlighted in bold.

**Fig 3:**
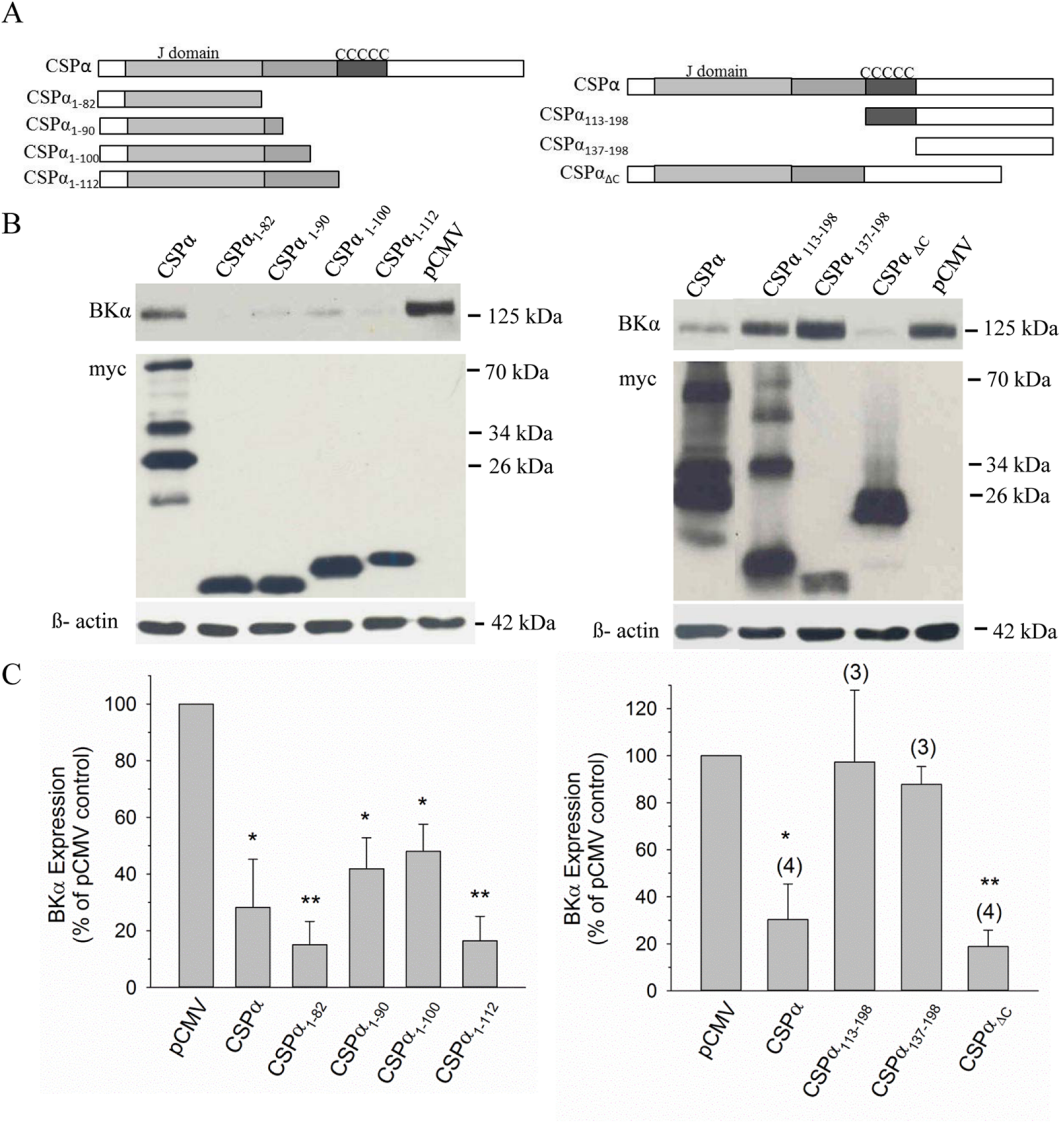
The J domain of CSPα reduces BK channel expression. **A.** Schematic of myc-tagged full length CSPα and CSPα deletion constructs. **B.** Western blot analysis of BK channel expression in CAD cells 24 h post-transfection with 1 μg cDNA encoding BKα subunit along with 0.75 μg myc-tagged full length CSPα cDNA or the indicated deletion constructs. As a transfection control, 0.75 μg empty pCMV was co-transfected with BKα subunit cDNA. 30 μg of cell lysate isolated under each experimental condition was separated by SDS-PAGE, probed with an anti-BKα subunit antibody and an anti-myc antibody. **Data shown in 3B right panel is from the same blot; a lane between lanes 1 and 2 was removed.** The histograms in panel **C** quantify changes in BK channel expression in the presence of wild-type CSPα and individual CSPα deletion mutants. Statistically significant differences from the pCMV control (set to 100%) were determined by one-way ANOVA; *p<0.05; **p<0.001.

## Supporting Information

S1 Blot Uncropped blots for Fig 1A, Fig 1C, and Fig 3B. (PPTX)
