# Supplementary material for: Correction: Cysteine String Protein Limits Expression of the Large Conductance, Calcium-Activated K+ (BK) Channel
Source: PLoS One. 2015 Oct 2;10(10):e0140073. doi: 10.1371/journal.pone.0140073 (PMC4591990; doi:10.1371/journal.pone.0140073)
Supplement: S1 Blot — (PPTX) [file pone.0140073.s001.pptx]

## Slide 1
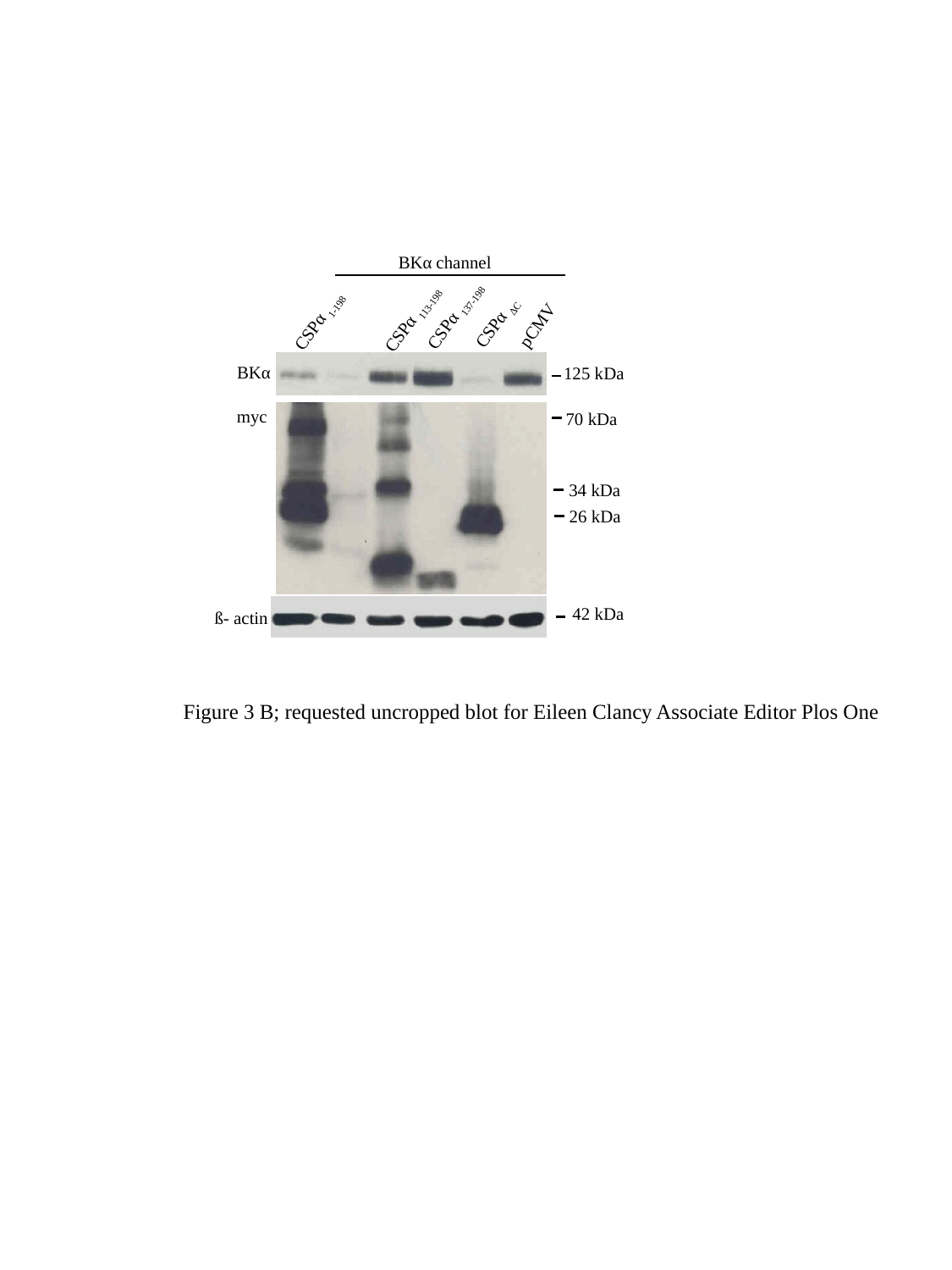

BKα channel
pCMV
CSPα 1-198
CSPα 137-198
CSPα 113-198
CSPα ΔC
BKα
125 kDa
myc
70 kDa
34 kDa
26 kDa
42 kDa
ß- actin
Figure 3 B; requested uncropped blot for Eileen Clancy Associate Editor Plos One

## Slide 2
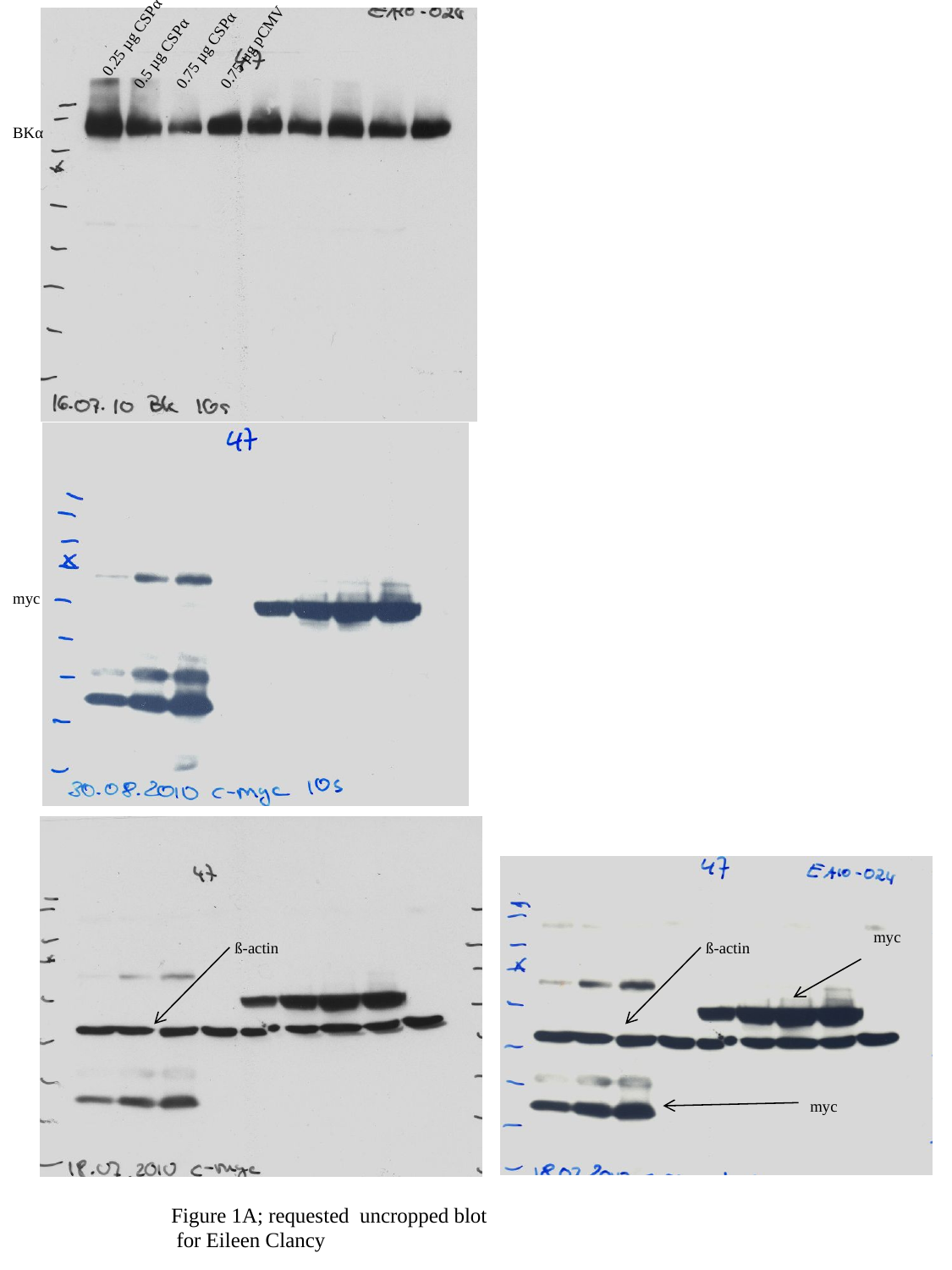

0.25 µg CSPα
0.75 µg pCMV
0.5 µg CSPα
0.75 µg CSPα
BKα
myc
myc
ß-actin
ß-actin
myc
Figure 1A; requested uncropped blot for Eileen Clancy

## Slide 3
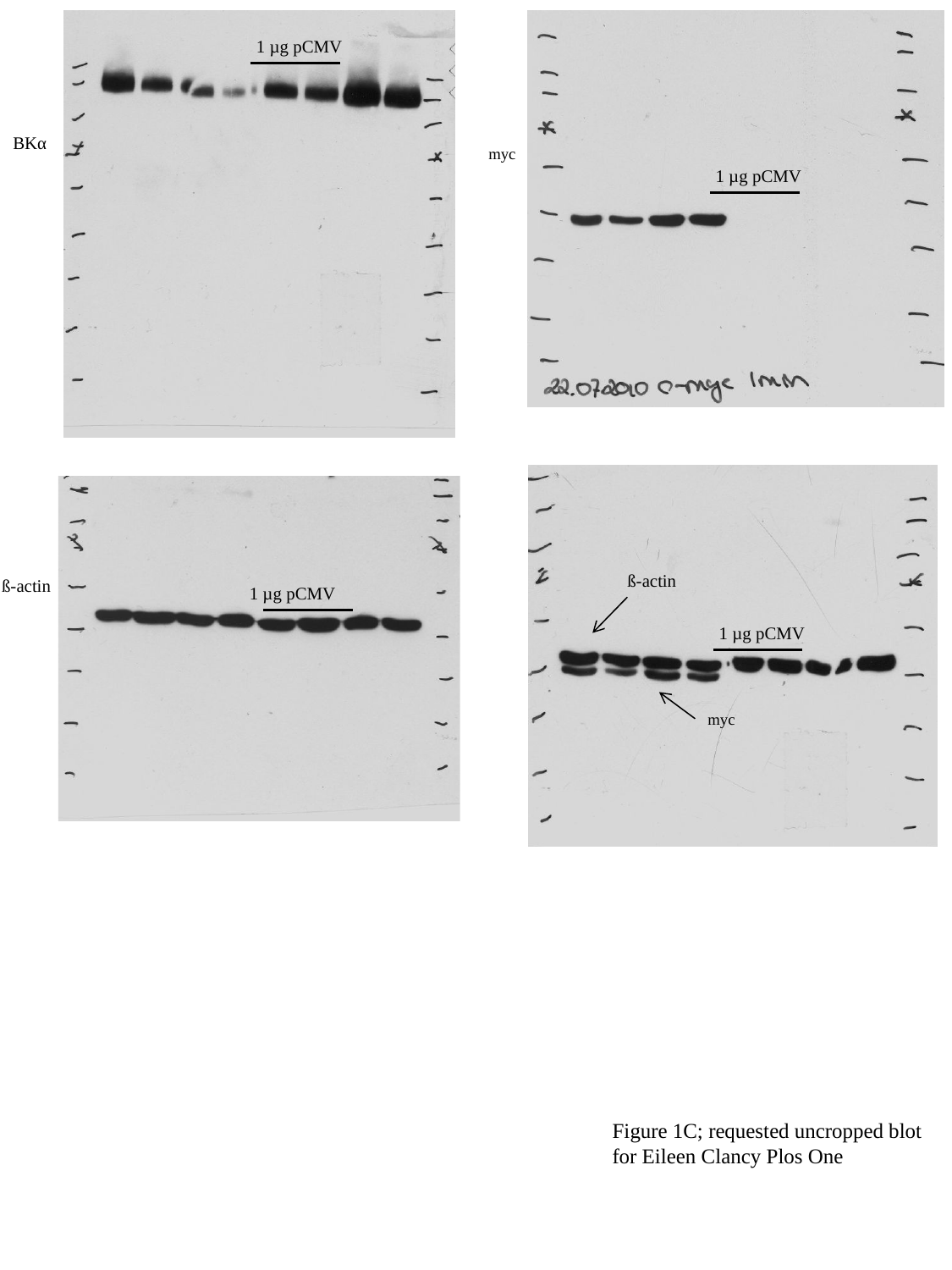

1 µg pCMV
BKα
myc
1 µg pCMV
ß-actin
ß-actin
1 µg pCMV
1 µg pCMV
myc
Figure 1C; requested uncropped blot for Eileen Clancy Plos One
